# Urban population exposure to air pollution in Europe over the last decades

**DOI:** 10.1186/s12302-020-00450-2

**Published:** 2021-03-07

**Authors:** Pierre Sicard, Evgenios Agathokleous, Alessandra De Marco, Elena Paoletti, Vicent Calatayud

**Affiliations:** 1 ARGANS, 260 route du Pin Montard, Biot, France; 2 Institute of Ecology, Key Laboratory of Agro-Meteorology of Jiangsu Province, School of Applied Meteorology, Nanjing University of Information Science and Technology, Nanjing, China; 3 Italian National Agency for New Technologies, Energy and the Environment, C.R. Casaccia, Italy; 4 Institute of Research On Terrestrial Ecosystems, National Research Council, Sesto Fiorentino, Italy; 5 Fundación CEAM, C/ Charles R. Darwin, Parque Tecnológico14, Paterna, Spain

**Keywords:** Air pollution, EU-28, Mann–kendall test, Population exposure, Risk assessment, Trend

## Abstract

**Background:**

The paper presents an overview of air quality in the 27 member countries of the European Union (EU) and the United Kingdom (previous EU-28), from 2000 to 2017. We reviewed the progress made towards meeting the air quality standards established by the EU Ambient Air Quality Directives (European Council Directive 2008/50/EC) and the World Health Organization (WHO) Air Quality Guidelines by estimating the trends (Mann-Kendal test) in national emissions of main air pollutants, urban population exposure to air pollution, and in mortality related to exposure to ambient fine particles (PM_2.5_) and tropospheric ozone (O_3_).

**Results:**

Despite significant reductions of emissions (e.g., sulfur oxides: ~ 80%, nitrogen oxides: ~ 46%, non-methane volatile organic compounds: ~ 44%, particulate matters with a diameter lower than 2.5 µm and 10 µm: ~ 30%), the EU-28 urban population was exposed to PM_2.5_ and O_3_ levels widely exceeding the WHO limit values for the protection of human health. Between 2000 and 2017, the annual PM_2.5_-related number of deaths decreased (- 4.85 per 10^6^ inhabitants) in line with a reduction of PM_2.5_ levels observed at urban air quality monitoring stations. The rising O_3_ levels became a major public health issue in the EU-28 cities where the annual O_3_-related number of premature deaths increased (+ 0.55 deaths per 10^6^ inhabitants).

**Conclusions:**

To achieve the objectives of the Ambient Air Quality Directives and mitigate air pollution impacts, actions need to be urgently taken at all governance levels. In this context, greening and re‐naturing cities and the implementation of fresh air corridors can help meet air quality standards, but also answer to social needs, as recently highlighted by the COVID-19 lockdowns.

## Background

Outdoor air pollution is a major global public health issue [48], leading to 4.2 million premature deaths worldwide [74] and half a million in the European Union (EU) in 2016 [24]. The EU identifies seven main air pollutants [45]: ammonia (NH_3_), nitrogen oxides (NO_x_), carbon monoxide (CO), particulate matter with an aerodynamic diameter lower than 2.5 µm and 10 µm (PM_2.5_ and PM_10_), sulfur oxides (SO_x_), tropospheric ozone (O_3_), and non‐methane volatile organic compounds (NMVOCs). In cities, where 74% of the EU population lives [33], PM_2.5_ and ground-level O_3_ have potentially the most significant effects on human health associated with respiratory and cardiovascular diseases and mortality, compared to other air pollutants [9, 55, 75]. In 2016, 374,000 and 14,600 non-accidental premature deaths were attributed to air pollution (PM_2.5_ and O_3_, respectively) in the EU-28^1^ countries [24]. Air pollution also damages plant ecosystems [35, 49, 63], and surface O_3_ is considered as the most detrimental air pollutant in terms of effects on vegetation and biodiversity [1, 52, 63].

The legislated ambient air quality standards and the emission control policies (e.g., [10, 18, 77]) control emissions of harmful substances into the atmosphere, and regulate the concentrations of air pollutants such as PM_2.5_, PM_10_, NO_2_ and O_3_, by setting limit and target values for the protection of human health Table 1 and requirements to ensure that Member States adequately monitor air quality in a harmonised manner. Therefore, the number of air quality monitoring stations grew rapidly in Europe, by an order of magnitude in 1996, with databases gathering air quality data such as the AirBase system of the European Environment Agency. The number of urban and suburban monitoring stations in Europe ranged from 1300 in 1990 to 3600 in 2000 and about 5000 stations in 2020. Due to the spatial representativeness of monitoring stations and the duration of time series, the above database offers an unprecedented way for trends analysis, and peer-reviewed articles. The Clean Air Programme for Europe (CAPE), published by the European Commission in 2013, aims to improve air quality in Europe by 2030 and to reduce the number of premature deaths by half compared with 2005 [16].

**Table 1:** Examples of air quality standards for common air pollutants as given in the European Ambient Air Quality Directive (Directive 2008/50/EC) and World Health Organization Air Quality Guidelines (WHO AQG) for the protection of human health

Air pollutant	EU limit and target value (threshold in µg m^−3^)	WHO AQG (threshold in µg m^−3^)
PM_10_^a^	Annual mean (40)	Annual mean (20)
PM_10_^a^	Number of exceedance of 24-h mean (50)	Number of exceedance of 24-h mean (50)
PM_2.5_^b^	Annual mean (25)	Annual mean (10)
PM_2.5_^b^	–	Number of exceedance of 24-h mean (25)
O_3_^c^	Number of exceedance of maximum daily 8-h mean (120)	Number of exceedance of maximum daily 8-h mean (100)
NO_2_^d^	Annual mean (40)	Annual mean (40)
NO_2_^d^	Number of exceedance of 1-h mean (200)	Number of exceedance of 1-h mean (200)
SO_2_^e^	Number of exceedance of 24-h mean (125)	Number of exceedance of 24-h mean (20)
CO^f^	Maximum daily 8-h (10,000)	Maximum daily 8-h (10,000)

^a^Annual mean PM_10_ concentration and number of days with 24-h PM_10_ concentration over 50 µg m^–3^ for the protection of human health. The annual mean PM_10_ concentration does not to exceed 40 µg m^–3^ (Directive 2008/50/EC) or 20 µg m^–3^ (WHO AQG). The 24-h PM_10_ mean concentration does not to exceed 50 µg m^–3^ (WHO AQG) or more than 35 times a year (EC)

^b^Annual mean PM_2.5_ concentration and numbers of days with 24-h PM_2.5_ mean concentration over 25 µg m^−3^ (WHO AQG). The annual mean PM_2.5_ concentration does not to exceed 25 µg m^–3^ (EC) or 10 µg m^–3^ (WHO AQG)

^c^For the protection of human health, the Directive 2008/50/EC has introduced a threshold of 120 µg m^–3^ for the daily maximum 8-h average. The threshold level should not be exceeded on more than 25 times a year. Number of days with daily maximum 8-h O_3_ concentrations over 100 µg m^–3^ as limit value for the protection of human health (WHO AQG)

^d^Annual mean NO_2_ concentration and number of hours with NO_2_ concentrations above 200 µg m^−3^. The annual mean NO_2_ concentration does not to exceed 40 µg m^–3^ (EC and WHO AQG) while the hourly threshold should not be exceeded more than 18 times a year (EC)

^e^The 24-h SO_2_ mean concentration does not to exceed 125 µg m^–3^ more than 3 times a year (EC) and does not to exceed 20 µg m^–3^ (WHO AQG). ^f^ The Directives have introduced a threshold of 10 mg m^–3^ for the maximum daily 8-h mean concentration

For the first time, through an extensive literature review and trends analysis, this study aims to (i) quantify the annual trends in national emissions of main air pollutants in the EU-28 countries over the time period 2000–2017, (ii) analyze the trends in real-world air pollutants concentrations over the last two decades; (iii) assess the effectiveness of emissions control policies for reducing the exposure of EU-28 population to ambient air pollution, and (iv) evaluate the impact of control policies on the number of premature deaths attributed to exposure to ambient PM_2.5_ and O_3_ levels over time.

## Materials and methods

### Data collection

The official national emissions of main air pollutants (SO_*x*_, NH_3_, PM_2.5_, PM_10_) and main O_3_ precursors (NO_x_, NMVOCs, CO), submitted by the Parties to the LRTAP Convention, were obtained through the Centre on Emission Inventories and Projections (CEIP) under the European Monitoring and Evaluation (EMEP) Program.^2^ The EU-28 urban population exposure was estimated by the European Environmental Agency (EEA) from data reported in Airbase, and the number of premature deaths attributed to exposure to ambient PM_2.5_ and O_3_ (per 10^6^ inhabitants) were obtained by the Organization for Economic Co-operation and Development^3^ (OECD). The above datasets were obtained over the time period 2000–2017.

### Estimation of urban population exposure

For each city included in the Urban Audit,^4^ the EU-28 urban population exposure to air pollutants above the EU limit values and WHO AQG was estimated by combining the concentration maps, from measured concentrations at urban and suburban background monitoring stations with more than 75% of validated hourly data per year, with the population density, and considering that the entire population is potentially exposed to the averaged concentrations, i.e., excluding human mobility [22–32]. The estimation of population exposure was based on data from about 1300 stations in 2000 to 3100 stations in 2017 in EU-28 countries.

### Estimation of the national number of premature deaths

The number of non-accidental premature deaths attributable to ambient PM_2.5_ and O_3_ were estimated for each EU member country and year by the method described in detail in Global Burden of Diseases [36] and widely used for the health risk assessment of air pollution [2, 3, 12, 37, 42–44, 61].

WHO set daily maximum 8-h concentrations for O_3_ and 24-h average concentration for PM_2.5_ as metrics to represent the mean daily exposure of population [76]. The daily population exposure to O_3_ and PM_2.5_ is estimated by combining concentrations maps from satellite and modeled data, and calibrated by ground measurements, with epidemiological data including relative risk values and baseline incidence rates [36]. For a health endpoint, the number of cases *NC*_*c*_ attributed to the exposure to the air pollutant *c* is calculated as NC_c_ $=$ BI $\times$ AP where BI is the baseline incidence rates and AP the attributable proportion, i.e., the fraction of a health endpoint that can be related to the exposure to *c* in a population *P*_*c*_ where RR is the relative risk value, i.e., the probability of developing a disease associated to an increase of 10 μg m^−3^ of the air pollutant *c* concentration [73].

1 $$\mathrm{AP}=\frac{\sum { [(RR}_{c}-1) \times {P}_{c}]}{\sum [{RR}_{c} \times {P}_{c}]}$$

The demographic data were taken from Eurostat [34], and the mortality data and BI were obtained by WHO [72]. The RR values were obtained from exposure–response functions, based on epidemiological studies, following recommendations from the Health Risks of Air Pollution in Europe project, and published by WHO [75]. For the non-accidental mortality (all ages), RR = 1.0123 and RR = 1.0029 are reported for PM_2.5_ and O_3_, respectively, i.e., for instance, a 10 μg m^−3^ increase in the 24-h average PM_2.5_ concentration is associated with a 1.2% increase in the risk for mortality attributed to non-accidental causes. However, the use of RR values and BI data from local (or national) epidemiological studies is recommended to obtain robust results.

### Statistical estimation of annual trends

A 10-year time-series is considered long enough to assess short-term changes [66]. The non-parametric Mann–Kendall test and the non-parametric Sen’s slope estimator were used to detect changes within time-series and estimate the magnitude of trends [38, 65]. Both tests were applied for annual national emissions of main air pollutants and the number of premature deaths attributed to exposure to ambient PM_2.5_ and O_3_ levels in EU-28 countries over the time period 2000–2017. In this study, we used MAKESENS program version 1.0 [56]. Results were considered significant at *p* < 0.05.

### Literature review

To report robust short-term air pollutants changes over the last 2 decades, approximately 50 peer-reviewed articles and technical report spanning over the time period 2000–2017 were retrieved from literature databases (Science Direct, Web of Science, and Google scholar). We selected the studies with: (i) in-situ observations from air quality monitoring networks (excluding modeled data); (ii) annual mean concentrations; (iii) at least 10-year time-series of data; (iv) more than 75% of data coverage annually; and (v) significant trend, i.e., with a *p* value < 0.05.

## Results and discussion

### Trends in national emissions

**Fig. 1:**
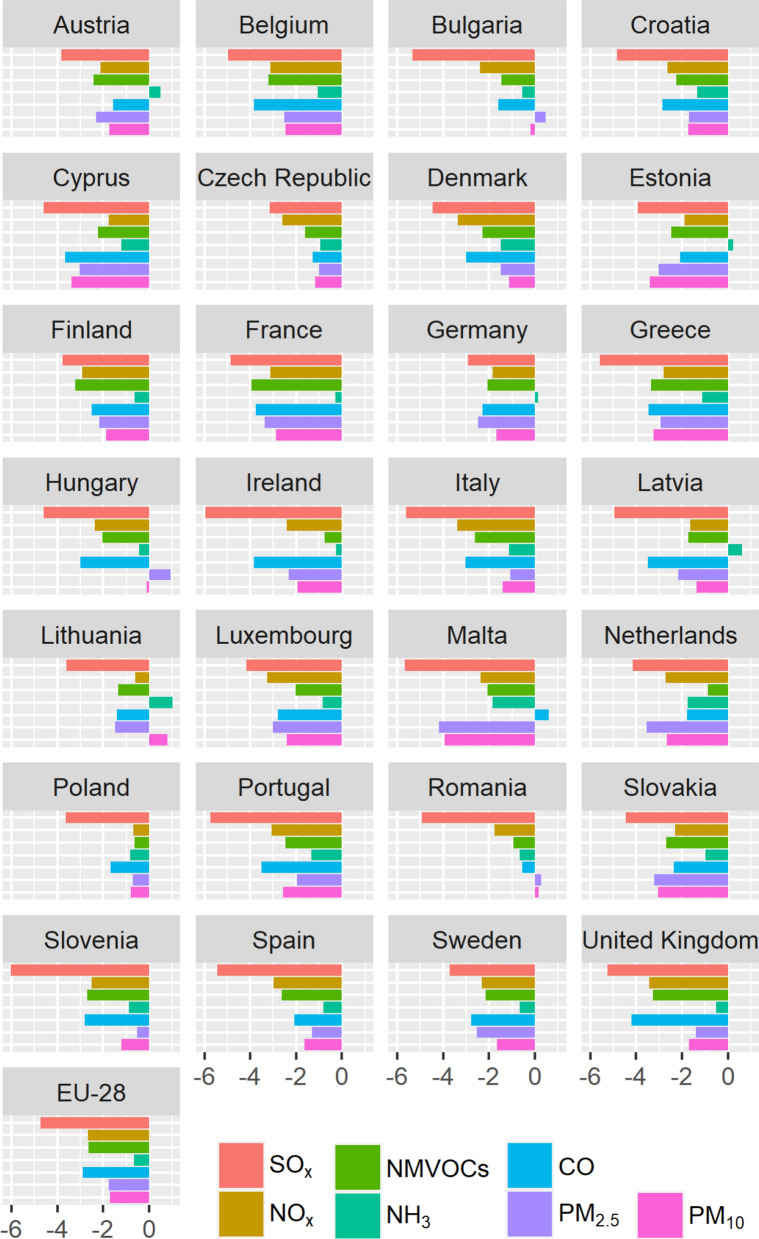
Annual trends of national emissions (% year^−1^) in the 28 European Union countries (EU-28) for sulfur oxides (SO_*x*_), nitrogen oxides (NO_*x*_), non-methane volatile organic compounds (NMVOCs), ammonia (NH_3_), carbon monoxide (CO), particulate matter with an aerodynamic diameter lower than 2.5 µm and 10 µm (PM_2.5_ and PM_10_) over the time period 2000–2017 (see Additional file 1: Table S1 for raw data). All trends are significant at *p* < 0.05 (Mann–Kendall)

Significant reductions were observed for the emission of all primary pollutants, i.e., − 4.7% year^−1^ for SO_*x*_, − 2.7% year^−1^ for NO_*x*_, − 2.6% year^−1^ for NMVOCs, − 0.6% year^−1^ for NH_3_, − 2.9% year^−1^ for CO and− 1.8% year^−1^ and− 1.7% year^−1^ for PM_2.5_ and PM_10_, respectively, over the time period 2000–2017 in the EU-28 countries (Fig. 1). The SO_*x*_ emissions decreased in all EU-28 countries, from − 2.9% year^−1^ (Germany) to − 6.0% year^−1^ (Slovenia). For NO_*x*_, the highest decrease was observed in the United Kingdom (− 3.4% year^−1^), while the lowest reduction was found in Lithuania (− 0.6% year^−1^) and Poland (− 0.7% year^−1^). For NMVOCs, the decrease ranged from − 0.6% year^−1^ (Poland) to − 4.0% year^−1^ (France). In general, small reductions were exhibited in the agricultural sector, contributing to 92% of NH_3_ emissions [22], but an increase could be determined in Austria, Estonia, Germany, Latvia and Lithuania, ranging from 0.1 to 1.0% per year. The domestic heating represents 48% of CO emissions [22]. Also, the CO emissions usually decreased, except in Malta (+ 0.6% year^−1^). A decrease of PM_2.5_ emissions was observed in all EU-28 countries, except Bulgaria (+ 0.5% year^−1^), Hungary (+ 0.9% year^−1^) and Romania (+ 0.3% year^−1^), associating with a slighter reduction in PM_10_ emissions (− 0.2% year^−1^ in Bulgaria, − 0.1% year^−1^ in Hungary). An increase of PM_10_ emissions was noted in Lithuania (+ 0.8% year^−1^) and Romania (+ 0.1% year^−1^). The highest decrease for PM_2.5_ (− 4.2% year^−1^) and PM_10_ (4.0% year^−1^) emissions occurred in Malta (Fig. 1).

The emissions of all primary air pollutants contributing to ambient levels of PM, O_3_, and NO_2_ decreased between 2000 and 2017 in the EU-28 (observed reductions SO_*x*_: − 80%; NO_*x*_: − 46%; NMVOCs: − 44%; NH_3_: − 10%; CO: − 49%; PM_2.5_: − 31%; PM_10_: − 29%), in line with stringent EC Directives, e.g. Air Quality Framework Directive [21], Large Combustion Plant Directive [19], and National Emission Ceilings Directives [17, 20], setting emission reduction commitments by 2030 compared to 2005 (expected reductions SO_2_: − 79%, NO_*x*_: − 63%, NMVOCs: − 40%, NH_3_: − 19%; PM_2.5_: − 49%). The emission reductions were mainly achieved as a result of the progress in e.g. the use of flue-gas abatement techniques, energy production and distribution, storage and distribution of solvents [28, 71], and vehicle technologies related to legislative “Euro” standards [59].

In EU-28 countries, the “on-road transport” sector is the largest contributor to total NO_x_ emissions (road transport: 40–55%), and represents 8–15% of VOCs emissions [22]. Diesel-powered motor vehicles account for about 91% of the fleet (from 81% in Czech Republic to 99% in Portugal) in all EU countries except for Greece (37%), and gasoline-powered motor vehicles account for about 7% of the fleet [41]. The Euro-2 to Euro-6 standards for light-duty vehicles were enforced from 1997 to 2015. For diesel cars, the average NO_*x*_ + VOCs limit ranged from 0.70 g/km (Euro-2) to 0.17 g/km (Euro-6), from 1.00 g/km to 0.50 g/km for CO and from 0.08 g/km to 0.0045 g/km for PM. For gasoline cars, the average NO_*x*_ + VOCs limit ranged from 0.500 g/km (Euro-2) and 0.128 g/km (Euro-6) and from 2.2 g/km to 1.0 g/km for CO. In 2017, the successive Euro standards have lowered the PM (94%), CO (50%) and NO_*x*_ + VOCs (76%) emission intensity in the EU compared to early 2000s. An investigation by Breuer et al. [7] in Germany showed that 91% of road transport NO_*x*_ emissions are produced by diesel-powered motor vehicles. At national level, emissions of NO_*x*_ from on-road transport decreased in all EU countries (from − 0.81% year^−1^ in Lithuania to − 4.29% year^−1^ in Finland) except in Poland (+ 1.51% year^−1^) and Romania (+ 1.17% year^−1^) between 2000 and 2017 (Additional file 1: Table S1). Investigations on NO_*x*_ emissions by diesel cars showed that, on average, their real-world NO_*x*_ emissions are seven times the limit of 0.08 g/km mandated by the Euro 6 standard [41]. Therefore, the reported reduction of NO_*x*_ emissions (− 46%) can be overestimated compared to the real-world NO_*x*_ emissions.

### Trends in urban population exposure

**Fig. 2:**
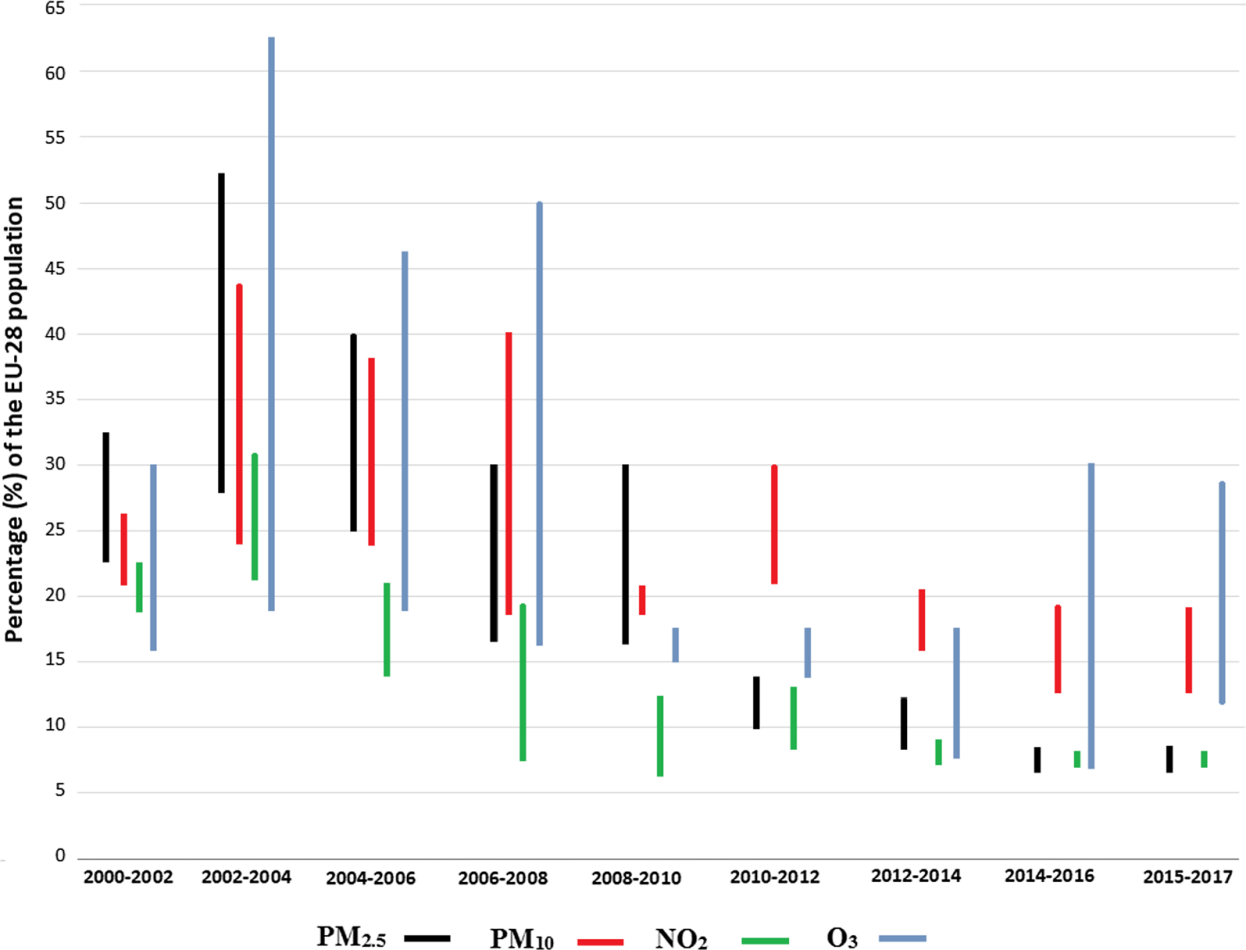
Minimum and maximum percentage of EU-28 population (in %) exposed to air pollutants concentrations (particulate matter PM_2.5_ and PM_10_, nitrogen dioxides NO_2_ and tropospheric ozone O_3_) exceeding the European Union limit or target values between 2000 and 2017 (see Additional file 1: Table S2; data source: [22–32]

Despite the reduction of PM_10_ emissions over the time period 2000–2017, the minimum and maximum percentage of the EU-28 urban population exposed to PM_10_ concentrations above the EU daily limit value ranged from 18 to 44% in 2000–2010 to 13–30% in 2010–2017 (Fig. 2), with the highest extent of exposure observed in 2003 (44%). Between 2000 and 2017, the EU daily limit value for PM_10_ was widely exceeded in Europe, mostly in Eastern Europe [38], e.g., Bulgaria, Cyprus, Czech Republic, Hungary, Poland, Slovakia, Greece, and Italy. In 2017, the EU daily limit value was exceeded in Bulgaria, Croatia, Czech Republic, Poland and Italy [22, 31]. Before 2006, more than 80% of the EU-28 population was exposed to PM_10_ levels exceeding the WHO AQG, reaching 42–52% in 2014–2017 (Additional file 1: Table S2). From 2000 to 2017, the annual averaged PM_10_ concentrations decreased by 0.65 μg m^−3^ year^−1^ on average at urban stations in the EU-28 [22]. In 2010–2017, 6–14% of the EU-28 population was exposed to PM_2.5_ levels above the EU annual target value, while the range was 16–52% in 2000–2010 (Fig. 2). The target value was exceeded mostly in Bulgaria, Czech Republic, Poland, and Slovakia between 2000 and 2013. The population exposure to PM_2.5_ levels above the WHO AQG ranged from more than 90% before 2006 to 74–80% in 2014–2017 Additional file 1: Table S2. Between 2000 and 2017, the annual averaged concentrations of PM_2.5_ decreased by on average 0.42 μg m^−3^ per year at urban background stations in the EU-28 [22].

The percentage of the EU-28 population exposed to NO_2_ concentrations above the EU annual limit value and the WHO AQG decreased from 14 to 31% before 2006, with the maximum recorded in 2003, to less than 10% since 2012 (Fig. 2). The annual limit value was mostly exceeded in Italy, Greece, and in the United Kingdom in 2000–2005, and in Germany in 2010–2016 [22–31]. The NO_2_ annual mean concentrations decreased by on average 0.39 μg m^−3^ year^−1^ over the time period 2002–2011 by joining 708 urban stations in the EU-28 [38]. The percentage of the EU-28 urban population exposed to SO_2_ levels above the EU daily limit value ranged from 1 to 2% in 2000–2005 to lower than 0.5% since 2007 (data not shown). The percentage of the EU-28 urban population exposed to SO_2_ levels exceeding the WHO AQG decreased from more than 70% before 2006 to less than 40% since 2013 [22–31]. Less than 2% of the EU-28 urban population was exposed to maximum CO daily 8-h mean concentrations above the EU and the WHO AQG (data not shown). Only a few traffic stations in Bulgaria, Poland and Romania have reported exceedances of the SO_2_ and CO EU limit values over the time period 2000–2017 [22, 38].

The EU-28 urban population exposed to O_3_ levels above the EU target value for human health protection ranged from 7 to 62% since 2000 (Fig. 2), with the highest extent of exposure observed in 2003. As for NO_2_ and PM_10_, the maximum O_3_ concentrations were observed in 2003, due to extremely warm summer in Europe, with a heatwave occurred in August, and stagnant weather conditions leading to accumulation of air pollutants [70]. The EU target value was mostly exceeded in Southern Europe, where higher background O_3_ levels (annual mean > 30 ppb) are observed [65], such as Croatia, Cyprus, France, Greece, Italy, Slovenia, Spain, Malta, Portugal, but also in Austria, Hungary, Luxembourg, and Poland recently. More than 95% of the total EU-28 urban population was exposed to O_3_ levels exceeding the WHO AQG since 2000 (Additional file 1: Table S2). In the EU, the annual mean of daily O_3_ concentrations increased by on average 0.05 ppb year^−1^ at 260 urban stations over the time period 2000–2014 Table 2. The annual O_3_ mean concentrations increased by on average 0.34 ppb year^−1^ at more than 80% of urban stations between 2005 and 2014, except in the United Kingdom where a decrease (− 0.18 ppb year^−1^) was observed at 65% of urban stations [59]. In Germany, an increase of 0.18 ppb year^−1^ was reported at 79 urban stations over the time period 2005–2018 [59]. A significant increase in the annual O_3_ mean (on average, + 0.29 ppb year^−1^) was found at urban stations in Southern Europe between 2000 and 2010 [46, 65]. In France, an increase of + 0.14 ppb year^−1^ at 76% of urban stations was reported between 1999 and 2012 [64]. Despite an increasing fleet size, the reduction in NO_*x*_ and VOCs emissions since the early 1990s, due to the vehicle emission regulations, allowed a reduction in O_3_ peaks and high percentiles [11, 26, 62]. At EU-28 urban stations, a reduction in O_3_ annual mean of the maximum daily 8-h mean values (− 0.75 ppb year^−1^) was found over the time period 2000–2014 [26]. In Southern Europe, significant reductions in 98th percentile (− 0.51 ppb year^−1^) and hourly maximum (− 1.81 ppb year^−1^) values were found at urban stations between 2000 and 2010 [65]. Simpson et al. [68] found an increase of O_3_ concentrations of 0.1–0.4 ppb year^−1^ up to the 95th O_3_ percentile over the time period 1990–2009. The surface O_3_ levels are rising in cities in Europe from 2000 (e.g., [8, 47, 59, 64, 67, 78], mainly due to a reduced titration of O_3_ by NO [40, 59].

**Table 2:** National-averaged trends magnitude (ppb per year ± standard deviation) of annual ozone mean concentrations at urban and rural background monitoring stations in Europe

Countries	Time period	References	*n*	Urban stations
Europe	1995–2012	[78]	289	+ 0.27 ± 0.10
Austria	1995–2014	[62]	6	+ 0.17 ± 0.12
Belgium			2	+ 0.08 ± 0.15
Germany			60	+ 0.19 ± 0.06
Greece			3	+ 0.18 ± 0.50
Netherlands			5	+ 0.19 ± 0.11
Slovenia			2	+ 0.14 ± 0.08
Spain			12	+ 0.36 ± 0.24
Sweden			3	+ 0.37 ± 0.10
Switzerland			11	+ 0.28 ± 0.11
United Kingdom			12	+ 0.21 ± 0.12
France	1999–2012	[64]	179	+ 0.14 ± 0.19
France	2000–2010	[46, 65]	29	+ 0.10 ± 0.30
Greece			3	+ 0.41 ± 0.15
Italy			20	+ 0.04 ± 0.30
Portugal			8	+ 0.40 ± 0.33
Spain			14	+ 0.48 ± 0.53
Europe	2000–2014	[8]	260	+ 0.05 ± 0.13
Belgium	2005–2014	[59]	2	+ 0.42 ± 0.05
France			136	+ 0.31 ± 0.42
Germany			79	+ 0.09 ± 0.17
Greece			4	+ 0.85 ± 0.43
Italy			50	+ 0.43 ± 0.84
Portugal			2	+ 0.48 ± 0.12
Spain			77	+ 0.54 ± 0.73
United Kingdom			29	- 0.18 ± 0.34
Germany	2005–2018	[59]	79	+ 0.18 ± 0.15

The studies were selected for more than 10-year time-series of ozone data, for stations with at least 75% of validated hourly data over the time period, and with a significant trend, i.e., with a *p* value < 0.05. Number of stations (*n*, with *n* ≥ 2)

### Trends in national mortality from exposure to ambient PM_2.5_ and *O*_*3*_ levels

**Fig. 3:**
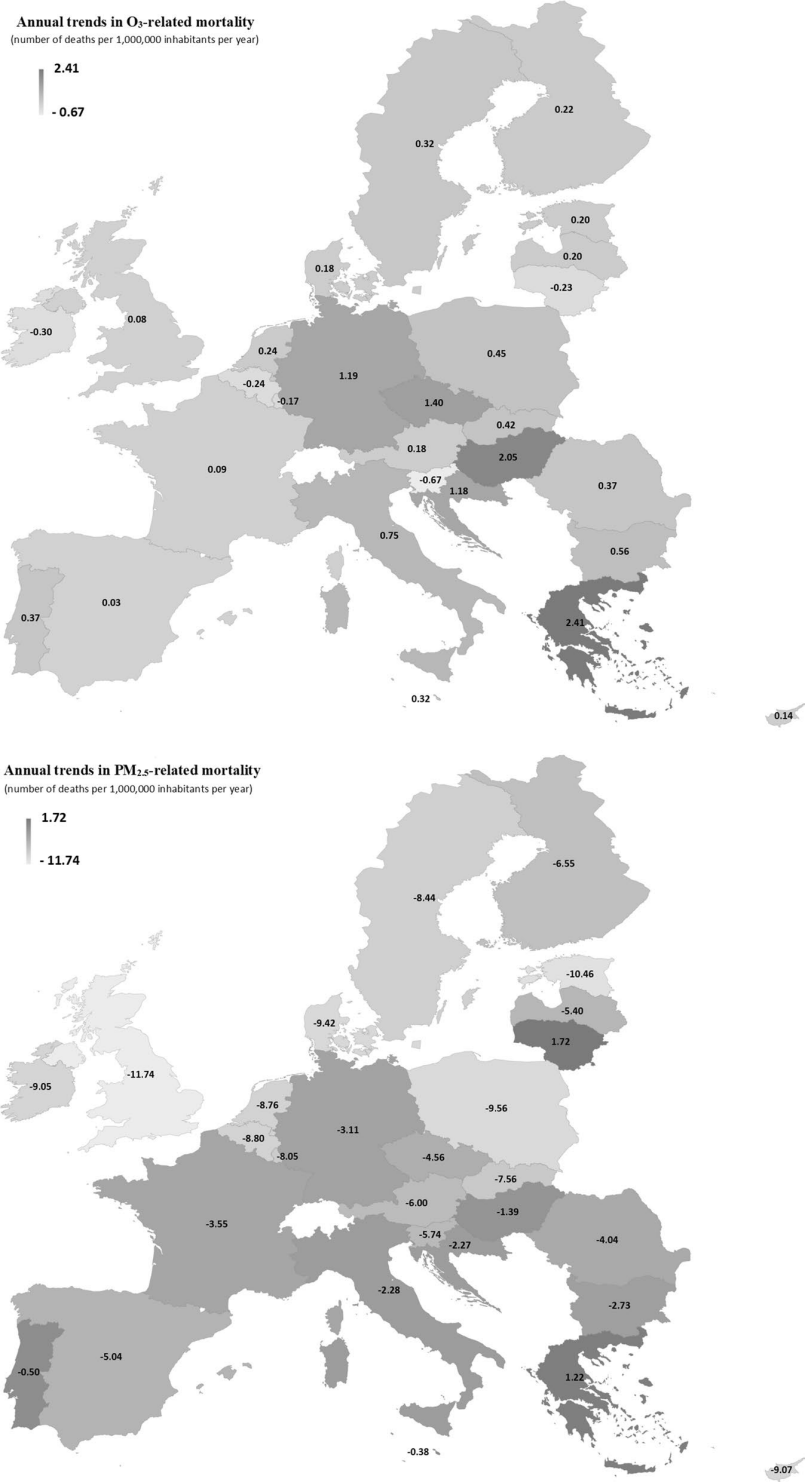
Annual trends of mortality (number of deaths per 1,000,000 inhabitants per year) due to ambient particulate matter with an aerodynamic diameter lower than 2.5 µm (PM_2.5_) and tropospheric ozone (O_3_) over the time period 2000–2017 in the 28 European Union countries (EU-28). Points below the thick line show a decrease in O_3_- and PM_2.5_-related mortality, while points above the thick line show an increase (see Additional file 1: Table S3 for raw data)

At present compared to other air pollutants, PM_2.5_ poses the most serious health risk in the EU-28 cities, associated with premature deaths and increased morbidity, followed by ground-level O_3_ [9, 55]. In the EU-28, the number of deaths due to ambient PM_2.5_ levels decreased by on average 4.85 per 1,000,000 inhabitants annually between 2000 and 2017 (Fig. 3). The highest annual decreases were observed in the United Kingdom and Estonia (− 11.74 and − 10.46 deaths per 10^6^ inhabitants, respectively) while a slighter reduction was found in Portugal (− 0.50 deaths per 10^6^ inhabitants). In Greece and Lithuania, an increase of annual mortality due to ambient PM_2.5_ levels was observed (+ 1.22 and + 1.72 deaths per 10^6^ inhabitants, respectively). In line with rising O_3_ levels in cities [59, 62], the annual O_3_-related number of premature deaths increased in the EU-28 (on average + 0.55 deaths per 10^6^ inhabitants). The highest annual decrease of mortality was observed in Greece (+ 2.41 deaths per 10^6^ inhabitants), Hungary (+ 2.05 deaths per 10^6^ inhabitants) and Czech Republic (+ 1.40 deaths per 10^6^ inhabitants), while a non-significant increase was found in Spain (+ 0.03 deaths per 10^6^ inhabitants). Between 2000 and 2017, the annual number of deaths attributed to O_3_ declined mostly in Northern Europe (e.g., Belgium: − 0.24, Ireland: − 0.30, Lithuania: − 0.23 deaths per 10^6^ inhabitants per year) where lower background O_3_ levels (annual mean < 20 ppb) were observed [4, 59]. In this study, only the outdoor exposure to air pollution was considered while people spend about 80–90% of time in indoor environments [54]. As the spatio-temporal variability of air pollutants levels and human mobility were ignored, the individual exposure estimates are slightly biased.

## Conclusions

Between 2000 and 2017, the EU-28 emissions fell for SO_*x*_ by about 80%, NO_*x*_: 46%, NMVOCs: 44%, NH_3_: 10%, CO: 49%, PM_2.5:_ 31%, and PM_10_: 29%. This confirms successful control strategies of air pollutants emissions. However, the current levels of air pollutants in cities continue to exceed the EU standards and WHO AQG for the protection of human health in Europe, especially for the secondary air pollutant O_3_ [12, 23, 38, 61]. In 2015–2017, the percentages of EU-28 urban population exposed to concentrations exceeding the WHO limit values were 74–81% for PM_2.5_, 42–52% for PM_10_, 95–98% for O_3_, 21–31% for SO_2_ and 7–8% for NO_2_ [22]. In agreement with a reduction of ambient PM_2.5_ levels in cities, the annual PM_2.5_-related number of deaths decreased (− 4.85 per 10^6^ inhabitants) between 2000 and 2017. The control strategies of O_3_ precursor emissions were effective in rural areas [53, 65]. However, the rising O_3_ levels have become a major public health issue in the EU-28 cities [47, 59, 62], where the annual O_3_-related number of premature deaths increased (+ 0.55 deaths per 10^6^ inhabitants) over the time period 2000–2017.

Barmpadimos et al. [5] have reported a positive correlation between PM_10_ and air temperature in summer (e.g., higher emissions from agriculture), and negative in winter (e.g., lower emissions by tertiary sector for heating). In Europe, the average annual air temperature increased by 0.22–0.40 °C per decade since 1965 [24]. The highest air temperature increase was observed over Eastern and Northern Europe in winter, and over Southern Europe in summer (EEA, 2018b). Climate change is projected to reduce the benefits of PM and O_3_ precursor emissions controls leading to higher PM and O_3_ levels.

There is an urgent need to take decisive actions at all governance levels to achieve the objectives of the Ambient Air Quality Directives as reported by the EC COM [15]. These actions span from improving air quality monitoring network, control of emission sources, improved mobility plans and raising awareness to citizens on the problem of air pollution, among others. In this context, urban and peri-urban reforestation and an implementation of fresh air corridors can help improve air quality and meet air quality standards in cities [6, 13, 51], but also answer to social needs, e.g., recreation, cultural, aesthetic [57, 58]. The cold air corridors are needed to reduce the climatic extreme events in large cities, which can lead to air pollution peaks.

Although outside the period of analysis, it is relevant to note that the recent COVID-19 pandemic could represent an opportunity for adopting measures that contribute to improve air quality in European cities in the future. Compared to the same period in 2017–2019, the lockdown measures in 2020 led to a decrease of NO (~ 63%) and NO_2_ (~ 52%) concentrations in Southern European cities due to the reduction of road and non-road transport [60, 69]. However, these measures did not significantly reduce the PM_2.5_ and PM_10_ levels (~ 8%) attributed to an increase of PM emissions from the activities at home (e.g., domestic heating, biomass burning), and during the lockdown, the ground-level O_3_ levels increased by ~ 17% due to a lower titration of O_3_ by NO [60]. While it is true that “*Air pollution rebounds in Europe’s cities as lockdowns ease*” (Financial Times, 24 June 2020) and that COVID discourages the use of public transport, there are some positive changes that, if sustained over time, might result in improvements of air quality in the cities in the future. Partial or total telework has been implemented in many companies and public offices, a change that will last to certain extent after the COVID pandemic reducing private car mobility. Cities like Barcelona and Paris have widened sidewalks to ensure social distancing on pedestrians, created more bicycle lanes and separated traffic and bus lanes for each direction.^5^

The COVID-19 lockdowns showed us the value of green urban spaces for our physical and mental wellbeing. Greening and re‐naturing cities are keywords of the EU Biodiversity Strategy for 2030 EC COM [14]. European Commission calls on European cities of at least 20,000 inhabitants to develop “*ambitious Urban Greening Plans”* by including the promotion of green infrastructure, nature-based solutions, and by planting at least 3 billion additional trees in the EU by 2030. Then, the COVID pandemic can be taken as an opportunity for the cities to foster changes in organization of the urban public space and re-think mobility [39], which hopefully may have relevant and lasting impacts on the quality of urban air. However, to efficiently reduce air pollution in cities, municipalities and city planners urgently need to base the selection of tree species upon quantitative and concrete assessments of the role of urban trees in affecting air quality either positively or negatively [62]. For improving air quality and thermal comfort in cities, tree planting programs need to: (a) plant and sustain healthy trees by selecting a diversity species well adapted to local conditions, (b) avoid species sensitive to air pollution, (c) use low VOCs and pollen emitting trees, (d) supply ample water to vegetation; (e) use long-lived and low maintenance species; and (f) implement cold air corridor in large cities to minimize the health risk of air pollutants [50, 62].

## Supplementary Information

**Additional file 1: Table S1.** Annual trends of national emissions (% year-1) in the 28 European Union countries (EU-28) for sulfur oxides (SOx), nitrogen oxides (NOx), on-road transport NOx (NOx_road), non-methane volatile organic compounds (NMVOCs), ammonia (NH3), carbon monoxide (CO), particulate matter with an aerodynamic diameter lower than 2.5 μm and 10 μm (PM2.5 and PM10) over the time period 2000–2017. All trends are significant at *p* < 0.05 (Mann-Kendal). The increasing trends are in bold. **Table S2.** Minimum and maximum percentage of EU-28 population (in %) exposed to air pollutants concentrations (tropospheric ozone O_3_, nitrogen dioxides NO_2_, particulate matter PM2.5 and PM10) exceeding the European Union (EU) and World Health Organization Air Quality Guidelines (WHO AQG) limit or target values between 2000 and 2017. **Table S3.** Annual trends of mortality (number of deaths per 1,000,000 inhabitants per year) due to ambient particulate matter with an aerodynamic diameter lower than 2.5 μm (PM2.5) and tropospheric ozone (O_3_) over the time period 2000–2017 in the 28 European Union countries (EU-28) with associated significance level *p* (Mann-Kendal *** *p* < 0.001; ** *p* < 0.01; * *p* < 0.05; + *p* < 0.1 and *p* < 0.1).

## Data Availability

Not applicable.

## Abbreviations

AP: Attributable proportion
AQG: Air Quality Guidelines
BI: Baseline incidence
CAPE: The Clean Air Programme for Europe
CLRTAP: Convention on Long-range Transboundary Air Pollution
CO: Carbon monoxide
EEA: European Environmental Agency
EMEP: European Monitoring and Evaluation Program
EU: European Union
GDB: Global Burden of Diseases
ICCT: International Council on Clean Transportation
*NC*_*c*_: Number of cases attributed to the exposure to the air pollutant *c*
NH_3_: Ammonia
NMVOCs: Non‐methane volatile organic compounds
NO_*x*_: Nitrogen oxides
O_3_: Tropospheric ozone
OECD: Organization for Economic Co-operation and Development
PM_10_: Particulate matter with an aerodynamic diameter lower than 10 µm
PM_2.5_: Particulate matter with an aerodynamic diameter lower than 2.5 µm
RR: Relative risk
SO_*x*_: Sulfur oxides
WHO: World Health Organization

## Footnotes

1 Including the United Kingdom, which withdrew from the European Union on 31st January 2020.

2 https://www.ceip.at.

3 https://stats.oecd.org.

4 https://ec.europa.eu/eurostat/web/cities/data/database.

5 http://www.xinhuanet.com/english/2020-05/19/c_139070452.htm; https://www.rfi.fr/en/france/20200520-france-bicycle-use-jumps-44-percent-since-end-coronavirus-confinement-paris-anne-hidalgo.
