# Supplementary material for: Urban population exposure to air pollution in Europe over the last decades
Source: Environ Sci Eur. 2021 Mar 7;33(1):28. doi: 10.1186/s12302-020-00450-2 (PMC7937352; doi:10.1186/s12302-020-00450-2)
Supplement: Supplementary file 1 — Additional file 1: Table S1. Annual trends of national emissions (% year-1) in the 28 European Union countries (EU-28) for sulfur oxides (SOx), nitrogen oxides (NOx), on-road transport NOx (NOx_road), non-methane volatile organic compounds (NMVOCs), ammonia (NH3), carbon monoxide (CO), particulate matter with an aerodynamic diameter lower than 2.5 μm and 10 μm (PM2.5 and PM10) over the time period 2000–2017. All trends are significant at p < 0.05 (Mann-Kendal). The increasing trends are in bold. Table S2. Minimum and maximum percentage of EU-28 population (in %) exposed to air pollutants concentrations (tropospheric ozone O3, nitrogen dioxides NO2, particulate matter PM2.5 and PM10) exceeding the European Union (EU) and World Health Organization Air Quality Guidelines (WHO AQG) limit or target values between 2000 and 2017. Table S3. Annual trends of mortality (number of deaths per 1,000,000 inhabitants per year) due to ambient particulate matter with an aerodynamic diameter lower than 2.5 μm (PM2.5) and tropospheric ozone (O3) over the time period 2000–2017 in the 28 European Union countries (EU-28) with associated significance level p (Mann-Kendal *** p < 0.001; ** p < 0.01; * p < 0.05; + p < 0.1 and p < 0.1). [file 12302_2020_450_MOESM1_ESM.docx]

Urban population exposure to air pollution in Europe over the last decades

Pierre Sicard^1^, Evgenios Agathokleous^2^, Alessandra De Marco^3^, Elena Paoletti^4^, Vicent Calatayud^5^

^1^ ARGANS, 260 route du Pin Montard, Biot, France; ^2^ Institute of Ecology, Key Laboratory of Agro-meteorology of Jiangsu Province, School of Applied Meteorology, Nanjing University of Information Science and Technology, Nanjing, China; ^3^ Italian National Agency for New Technologies, Energy and the Environment, C.R. Casaccia, Italy; ^4^ Institute of Research on Terrestrial Ecosystems, National Research Council, Sesto Fiorentino, Italy; ^5^ Fundación CEAM, Parque Tecnológico, C/ Charles R. Darwin, 14, Paterna, Spain.

Additional file

**Table S1 -** Annual trends of national emissions (% year^-1^) in the 28 European Union countries (EU-28) for sulfur oxides (SO_x_), nitrogen oxides (NO_x_), on-road transport NO_x_ (NO_x__road), non-methane volatile organic compounds (NMVOCs), ammonia (NH_3_), carbon monoxide (CO), particulate matter with an aerodynamic diameter lower than 2.5 µm and 10 µm (PM_2.5_ and PM_10_) over the time period 2000-2017. All trends are significant at *p* < 0.05 (Mann-Kendall). The increasing trends are in bold.

| **EU-28 Countries** | **SO_x_** | **NO_x_** | **NO_x__road** | **NMVOCs** | **NH_3_** | **CO** | **PM_2.5_** | **PM_10_** |
| --- | --- | --- | --- | --- | --- | --- | --- | --- |
| **Austria** | - 3.83 | - 2.11 | - 2.40 | - 2.41 | **+ 0.51** | - 1.57 | - 2.31 | - 1.74 |
| **Belgium** | - 4.98 | - 3.12 | - 3.00 | - 3.20 | - 1.07 | - 3.85 | - 2.53 | - 2.45 |
| **Bulgaria** | - 5.35 | - 2.41 | - 1.39 | - 1.47 | - 0.54 | - 1.59 | **+ 0.47** | - 0.19 |
| **Croatia** | - 4.84 | - 2.63 | - 2.41 | - 2.25 | - 1.34 | - 2.85 | - 1.71 | - 1.72 |
| **Cyprus** | - 4.58 | - 1.77 | - 2.56 | - 2.23 | - 1.22 | - 3.65 | - 3.03 | - 3.39 |
| **Czech Republic** | - 3.15 | - 2.60 | - 2.13 | - 1.60 | - 0.96 | - 1.27 | - 0.99 | - 1.16 |
| **Denmark** | - 4.45 | - 3.35 | - 3.67 | - 2.30 | - 1.48 | - 3.00 | - 1.49 | - 1.13 |
| **Estonia** | - 3.92 | - 1.88 | - 3.22 | - 2.48 | **+ 0.24** | - 2.07 | - 3.01 | - 3.41 |
| **Finland** | - 3.77 | - 2.92 | - 4.29 | - 3.23 | - 0.64 | - 2.51 | - 2.17 | - 1.88 |
| **France** | - 4.85 | - 3.12 | - 3.19 | - 3.96 | - 0.30 | - 3.75 | - 3.37 | - 2.87 |
| **Germany** | - 2.93 | - 1.86 | - 2.73 | - 2.07 | **+ 0.13** | - 2.29 | - 2.48 | - 1.67 |
| **Greece** | - 5.59 | - 2.79 | - 3.50 | - 3.36 | - 1.11 | - 3.46 | - 2.94 | - 3.25 |
| **Hungary** | - 4.59 | - 2.35 | - 2.68 | - 2.04 | - 0.44 | - 3.00 | **+ 0.93** | - 0.10 |
| **Ireland** | - 5.97 | - 2.40 | - 2.49 | - 0.75 | - 0.26 | - 3.84 | - 2.33 | - 1.94 |
| **Italy** | - 5.63 | - 3.40 | - 3.85 | - 2.63 | - 1.12 | - 3.03 | - 1.07 | - 1.40 |
| **Latvia** | - 4.94 | - 1.63 | - 2.83 | - 1.73 | **+ 0.61** | - 3.49 | - 2.16 | - 1.36 |
| **Lithuania** | - 3.60 | - 0.61 | - 0.81 | - 1.35 | **+ 1.01** | - 1.39 | - 1.49 | **+ 0.79** |
| **Luxembourg** | - 4.18 | - 3.25 | - 3.44 | - 2.02 | - 0.84 | - 2.80 | - 3.00 | - 2.42 |
| **Malta** | - 5.66 | - 2.37 | - 2.56 | - 2.06 | - 1.86 | **+ 0.60** | - 4.20 | - 3.95 |
| **Netherlands** | - 4.14 | - 2.72 | - 3.01 | - 0.88 | - 1.76 | - 1.78 | - 3.53 | - 2.65 |
| **Poland** | - 3.62 | - 0.69 | **+ 1.51** | - 0.62 | - 0.81 | - 1.67 | - 0.72 | - 0.79 |
| **Portugal** | - 5.73 | - 3.06 | - 3.07 | - 2.46 | - 1.34 | - 3.50 | - 1.96 | - 2.56 |
| **Romania** | - 4.94 | - 1.77 | **+ 1.17** | - 0.95 | - 0.67 | - 0.55 | **+ 0.28** | **+ 0.15** |
| **Slovakia** | - 4.46 | - 2.31 | - 1.71 | - 2.69 | - 0.98 | - 2.36 | - 3.22 | - 3.06 |
| **Slovenia** | - 6.03 | - 2.50 | - 1.15 | - 2.70 | - 0.89 | - 2.79 | - 0.52 | - 1.22 |
| **Spain** | - 5.44 | - 2.99 | - 3.22 | - 2.62 | - 0.82 | - 2.08 | - 1.30 | - 1.63 |
| **Sweden** | - 3.71 | - 2.32 | - 3.56 | - 2.15 | - 0.66 | - 2.77 | - 2.54 | - 1.64 |
| **United Kingdom** | - 5.26 | - 3.42 | - 4.07 | - 3.28 | - 0.52 | - 4.21 | - 1.40 | - 1.70 |
| **EU-28** | - 4.74 | - 2.67 | - 2.51 | - 2.63 | - 0.65 | - 2.89 | - 1.76 | - 1.70 |

**Table S2 -** Minimum and maximum percentage of EU-28 population (in %) exposed to air pollutants concentrations (tropospheric ozone O_3_, nitrogen dioxides NO_2_, particulate matter PM_2.5_ and PM_10_) exceeding the European Union (EU) and World Health Organization Air Quality Guidelines (WHO AQG) limit or target values between 2000 and 2017.

| **Time period** | **O_3_**  (Min-Max) | | **NO_2_**  (Min-Max) | | **PM_2.5_**  (Min-Max) | | **PM_10_**  (Min-Max) | |
| --- | --- | --- | --- | --- | --- | --- | --- | --- |
| *Target values* | *EU* | *WHO* | *EU* | *WHO* | *EU* | *WHO* | *EU* | *WHO* |
| **2000-2002** | 16-30 | > 95 | 19-22 | > 15 | 23-33 | > 90 | 21-26 | > 80 |
| **2002-2004** | 19-62 |  | 21-27 |  | 27-52 |  | 24-42 |  |
| **2004-2006** | 19-46 |  | 14-21 |  | 25-40 |  | 24-38 |  |
| **2006-2008** | 16-50 | > 95 | 7-19 | 7-19 | 16-31 | 92-95 | 18-40 | 80-90 |
| **2008-2010** | 15-17 | > 97 | 6-12 | 6-12 | 16-30 | 90-95 | 18-21 | 80-81 |
| **2010-2012** | 14-17 | 95-98 | 8-13 | 8-13 | 10-14 | 91-93 | 21-30 | 64-83 |
| **2012-2014** | 8-17 | 96-98 | 7-9 | 7-9 | 8-12 | 85-91 | 16-21 | 50-63 |
| **2014-2016** | 7-30 | 95-98 | 7-8 | 7-8 | 6-8 | 74-85 | 13-19 | 42-52 |
| **2015-2017** | 12-29 | 95-98 | 7-8 | 7-8 | 6-8 | 74-81 | 13-19 | 42-52 |

**Table S3 -** Annual trends of mortality (number of deaths per 1,000,000 inhabitants per year) due to ambient particulate matter with an aerodynamic diameter lower than 2.5 µm (PM_2.5_) and tropospheric ozone (O_3_) over the time period 2000-2017 in the 28 European Union countries (EU-28) with associated significance level *p* (Mann-Kendall *** *p* < 0.001; ** *p* < 0.01; * *p* < 0.05; + *p* < 0.1 and *p* > 0.1).

| **EU-28 Countries** | **PM_2.5_-related mortality** | ***p level*** | **O_3_-related mortality** | ***p level*** |
| --- | --- | --- | --- | --- |
| **Austria** | - 6.00 | *** | 0.18 | ** |
| **Belgium** | - 8.80 | *** | - 0.24 | * |
| **Bulgaria** | - 2.73 | * | 0.56 | *** |
| **Croatia** | - 2.27 | + | 1.18 | *** |
| **Cyprus** | - 9.07 | *** | 0.14 |  |
| **Czech Republic** | - 4.56 | *** | 1.40 | *** |
| **Denmark** | - 9.42 | *** | 0.18 |  |
| **Estonia** | - 10.46 | *** | 0.20 | *** |
| **Finland** | - 6.55 | *** | 0.22 | *** |
| **France** | - 3.55 | *** | 0.09 |  |
| **Germany** | - 3.11 | *** | 1.19 | *** |
| **Greece** | 1.22 | * | 2.41 | *** |
| **Hungary** | - 1.39 |  | 2.05 | *** |
| **Ireland** | - 9.05 | *** | - 0.30 | + |
| **Italy** | - 2.28 | ** | 0.75 | *** |
| **Latvia** | - 5.40 | * | 0.20 | *** |
| **Lithuania** | 1.72 | + | - 0.23 | *** |
| **Luxembourg** | - 8.05 | *** | - 0.17 | * |
| **Malta** | - 0.38 |  | 0.32 | *** |
| **Netherlands** | - 8.76 | *** | 0.24 | * |
| **Poland** | - 9.56 | *** | 0.45 | *** |
| **Portugal** | - 0.50 |  | 0.37 | *** |
| **Romania** | - 4.04 | *** | 0.37 | *** |
| **Slovakia** | - 7.56 | *** | 0.42 | *** |
| **Slovenia** | - 5.74 | *** | - 0.67 | *** |
| **Spain** | - 5.04 | *** | 0.03 |  |
| **Sweden** | - 8.44 | *** | 0.32 | *** |
| **United Kingdom** | - 11.74 | *** | 0.08 |  |
| **EU-28** | **- 4.85** | ******* | **0.55** | ******* |
